# Computational design of a 3D magnetic particle imaging (MPI) prototype

**DOI:** 10.1063/9.0000971

**Published:** 2026-03-06

**Authors:** Shahriar Mostufa, Bahareh Rezaei, Kai Wu

**Affiliations:** Department of Electrical and Computer Engineering, Texas Tech University, Lubbock, Texas 79409, USA

## Abstract

Magnetic particle imaging (MPI) is an emerging imaging modality that exploits the magnetization response of magnetic nanoparticle tracers. While MPI offers substantially higher resolution compared to magnetic resonance imaging, its translation to human-scale applications remains limited. These challenges stem from the requirement of high-intensity electric currents to generate strong magnetic fields, as well as reduced field uniformity with increasing coil spacing. To overcome these barriers, comprehensive simulation studies are essential for guiding MPI prototype design and performance optimization. In this work, we present a finite element method (FEM)-based design of a three-dimensional (3D) MPI prototype. The system integrates electromagnetic coils for the selection, drive, and focus fields, along with a gradiometer configuration for signal reception. Each coil’s geometry and magnetic field were first simulated independently to validate its ability to generate the desired magnetic field and subsequently combined into a full-system design with time-domain input excitation signals. This framework achieved 3D field-free point (FFP) scanning within a 20 mm^3^ field of view. The selection field provided a gradient of 4, 2, and 2 T/m in z axis, y axis, and x axis, respectively, the drive field produced 20 mT, and the focus fields generated 40 mT (z-axis) and 20 mT (y-axis), enabling controlled spatial movement of the FFP. Overall, this study establishes a complete 3D FEM simulation framework for MPI system design and lays the foundation for future optimization toward clinical-scale applications.

## I. INTRODUCTION

Magnetic particle imaging (MPI) is a non-ionizing biomedical imaging technique that employs the dynamic magnetization of magnetic nanoparticles (MNPs) to generate spatially resolved images.^1–6^ As a novel imaging modality, MPI offers superior sensitivity and resolution compared to conventional techniques such as magnetic resonance imaging (MRI).^7^ Unlike MRI, MPI directly detects the signal induced by MNPs through Faraday’s law of induction.^8–10^ Moreover, the tracers used in MPI are typically iron oxide–based, which are biocompatible and clinically safe. This represents a significant advantage over modalities such as X-ray, which exposes patients to harmful ionizing radiation, and MRI contrast agents such as iodine or gadolinium, which are unsuitable for patients with chronic kidney disease (CKD).^11–13^

Spatial encoding in MPI is achieved using a constant selection field that generates a field-free point (FFP). Drive fields and focus fields are then applied to move the FFP within the imaging volume.^14^ The drive field generates an alternating magnetic field (AMF) that is applied uniformly across the entire imaging volume. Due to the nonlinear magnetization response, only MNPs within the FFP contribute to a measurable signal. MNPs outside the FFP remain magnetically saturated by the selection field and thus, contribute no signal. The drive field inherently defines a limited field of view (FOV) region, which restricts imaging to a small object space and cannot accommodate larger regions without additional methods such as focus fields. Focus fields are employed to expand the FOV in three dimensions, enabling larger-scale imaging that cannot be achieved using drive coils alone. Despite these advantages, MPI has not yet reached clinical, human-scale application due to challenges in coil-based magnetic field generation. As the FOV increases, it becomes increasingly difficult to maintain both high field amplitude and uniformity. In addition, high power consumption and coil heating pose significant limitations for long-term clinical operation. To address these issues, several coil designs and system prototypes have been proposed. For instance, Irfan *et al.*^15^ proposed an finite element method (FEM)-based computational design of MPI selection field coils, incorporating hybrid configurations with permanent magnet to improve field generation up to 4 T/m with enhanced uniformity. Wei *et al.*^16^ proposed the first nonhuman primate-sized 3D MPI system with digital scanning, featuring an increased bore size of 190 mm. Wang *et al.*^17^ introduced a simulation design of a single-sided MPI system capable of scanning from the surface to a certain depth, thereby extending MPI imaging from a closed space to an open space. Shen *et al.*^18^ reported a 3D MPI framework using an open-source simulation software tool, focusing primarily on image generation from the MPI scanner. Meribout and Kalra^19^ demonstrated 2D MPI simulation of a Halbach array with a 20 mm^3^ FOV using FEM analysis. Nigam *et al.*^20^ applied machine learning and deep learning in the MPI system to enhance image reconstruction capabilities. Despite these advances, the literature still lacks a comprehensive 3D FEM-based electromagnetic coil design for MPI that integrates coil optimization with complete FFP trajectory scanning.

This paper presents a comprehensive computational study of an MPI system prototype, integrating electromagnetic coil design with complete 3D FFP scanning. FEM simulations were conducted in COMSOL Multiphysics to design Maxwell coil pairs for gradient fields, Helmholtz coils for focus fields, and solenoidal coils for drive, receive, and compensation functions. The study focused on a 20 mm^3^ object space, where the magnetic field amplitudes and uniformities were systematically evaluated. Finally, time-domain excitations were applied to the coil system to demonstrate controlled 3D movement of the FFP across the imaging volume, achieved through the coordinated interaction of the selection, drive, and focus fields.

## II. COMPUTATIONAL DESIGN OF MPI COILS

**FIG. 1.**
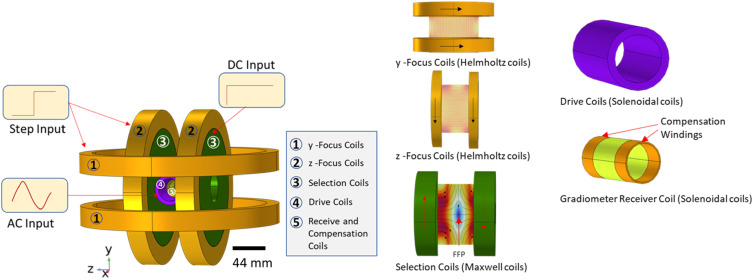
Schematic of the computational 3D model of the MPI prototype system in COMSOL for 3D scanning, showing selection-field coil pairs, focus-field coil pairs, drive-field coils, and gradiometer receiving coils.

We designed and analyzed the 3D scanning MPI system using COMSOL Multiphysics, a FEM–based computational analysis platform. Figure 1 presents the complete schematic of the 3D MPI system model. The geometry was created using the 3D Geometry module, while the AC/DC module’s *Magnetic Fields* interface was used to compute the magnetic fields generated by the coils. The coils were modeled using the *homogenized multi-turn* option, with circulating currents defined as time-dependent functions. Copper was used as the material for the electromagnetic coils, and air as the background medium. Infinite boundary conditions were applied to define the simulation domain.

**FIG. 2.**
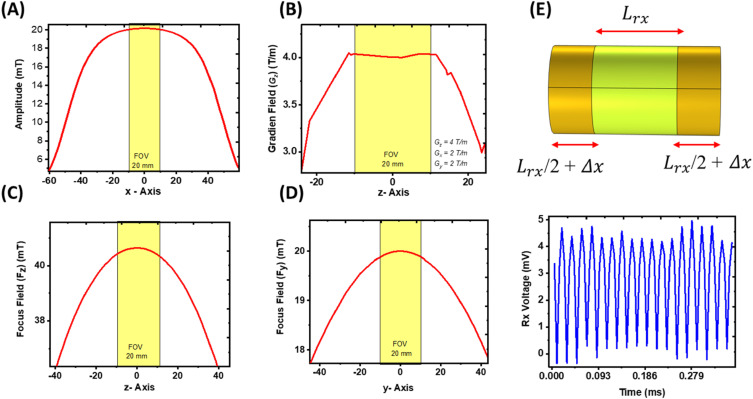
Magnetic field amplitudes and uniformity within the 20 mm^3^ FOV for (a) drive-field coil, (b) selection-field coils (z-axis), (c) z-axis focus coils, (d) y-axis focus coils, and (e) gradiometer receiving coils with compensated feedthrough signals.

The computational model was developed in a sequential manner, beginning with the design of the drive field coil (x-axis), which provides the primary excitation component of the MPI system. This coil was arranged in a solenoidal configuration and driven by an AC current at 25 kHz. A frequency-domain analysis was performed to achieve the target magnetic field amplitude ($H_{dx}$ = 20 mT) with uniformity across a 20 mm FOV_x_, as illustrated in Fig. 2(a). Iterative optimization of the coil geometry and electrical parameters was conducted to meet performance requirements. After the establishment of the drive field, gradiometer receiver coils (x-axis) incorporating compensation windings were designed and simulated. The receiver and compensation coils were fine-tuned to suppress feedthrough signals from the drive field, thereby improving detection sensitivity. To achieve this, the compensation coil height was varied, which in turn adjusted the winding number until the residual signal was minimized. Figure 2(d) shows the detailed gradiometer configuration, where the compensated feedthrough signal was reduced to ∼4 mV. This careful design enables reliable detection of the much weaker signals generated by the MNPs. This suppression of feedthrough signal is critical for achieving higher image quality and improved signal-to-noise ratio (SNR), enabling detection of the much weaker MNP-generated signals even at relatively low nanoparticle concentrations.

Next, the selection field coils (z-axis) were designed in a Maxwell configuration to generate the required gradient field with high uniformity across the 20 mm FOV. Owing to the opposing DC current directions in the coil windings, the magnetic fields canceled at the center, thereby creating the FFP. Using the *stationary study*, coil parameters were optimized to achieve a gradient strength of G_z_=4 T/m (with G_x_ = −2 T/m and G_y_ = −2 T/m) and uniformity across the FOV, as illustrated in Fig. 2(b).

The focus field coils were then implemented in a Helmholtz arrangement along the z- and y-axes to enable precise movement of the FFP within the imaging volume. These focus field coils were tuned to generate uniform magnetic fields along their respective axes: the z-axis coils generated H_fz_ = 40 mT within the 20 mm FOV_z_ [Fig. 2(c)], while the y-axis coils generated H_fy_ = 20 mT within the 20 mm FOV_y_ [Fig. 2(d)]. These field strengths were selected to complement the gradient field, allowing scanning across the full 20 × 20 × 20 mm^3^ imaging volume. The details of the electromagnetic coils’ structural dimensions are listed in Table I. The relationships between field amplitudes, gradients, and FOV are given by^21^

$$FOV_{x}=\frac{2H_{dx}}{G_{x}}=\frac{2\times 20\text{mT}}{2T/m}=20\text{mm,}$$ (1)

$$FOV_{y}=\frac{2H_{fy}}{G_{y}}=\frac{2\times 20\text{mT}}{2T/m}=20\text{mm,}$$ (2)

$$FOV_{z}=\frac{2{H_{f}}_{z}}{G_{z}}=\frac{2\times 40\text{mT}}{4T/m}=20\text{mm.}$$ (3)

In designing these coils, emphasis was placed on tuning each set of windings to achieve both the target magnetic field amplitudes and high uniformity across the FOV. Specifically, the system was engineered to maintain uniformity up to ∼95% of the maximum achievable level, ensuring consistent imaging performance. Additionally, to evaluate coil heating at the maximum scanning duration of 4.84 ms, we performed coupled electromagnetic Joule heating and heat dissipation simulations in air using the *heat transfer in solid* physics. Thus, the coils operate only for very short durations for this small size scanner; the maximum temperature rise (ΔT) observed in each selection-field coil, focus field z, and focus field y is only 0.001 °C, and almost negligible in the drive coils after continuous operation for a maximum scanning duration of 4.84 ms for single scan of the whole FOV with finer 2 mm resolutions. After optimizing each coil set individually, the full system was simulated with a *time-domain study* using a fine physics-controlled mesh (total degrees of freedom: 1 215 274).

**TABLE I.** Coil designs specifications.

Specifications	Selection coils (each)	Drive coils	z-focus coils (each)	y-focus coils (each)	Receiver coils	Compensation coils (each)
Inner diameter (mm)	40	30	154.96	229.38	22	22
Outer diameter (mm)	96.98	34.8	179.38	245.66	22.8	22.8
Height (mm)	25	97.68	25	25	22	11.187
Total windings (layers)	537.47 (35)	180 (3)	∼230 (15)	∼153 (10)	440 (4)	∼223 (4)
numbers						
Excitation current (A)	18	9.25	15.5	14.3	0	0
Wire diameter	1.628 (mm)	1.628 (mm)	1.628 (mm)	1.628 (mm)	0.2 (mm)	0.2 (mm)
and AWG	AWG-14	AWG-14	AWG-14	AWG-14	AWG-32	AWG-32
Resistance (R) ohm	1.31	0.157	1.03	0.948	16.7	8.5
Inductance (L)	16.353 (mH)	0.493 85 (mH)	14.043 (mH)	12.604 (mH)	2.9609 (mH 1A)	1.1362 (mH 1A)
Generated field	Gz = 4 T/m	20 mT	40 mT	20 mT	No	No
	Gy=Gx = −2 T/m					

**FIG. 3.**
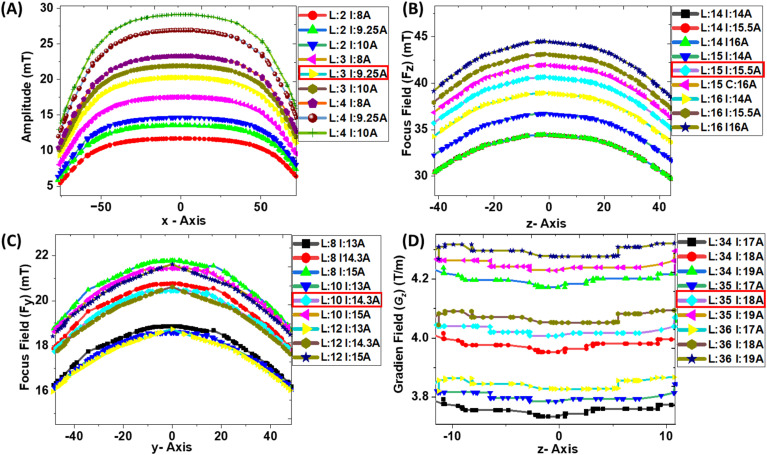
Optimization of the number of winding layers (L) and excitation currents (I) to achieve the required magnetic field for (a) the drive-field coil 20 mT, (b) the z-axis focus coils 40 mT, (c) the y-axis focus coils 20 mT, and (d) the z-axis selection-field coils 4 T/m with uniformity. The minimum windings and current required to generate the required magnetic field amplitude are also highlighted with red color box.

Another crucial factor considered during the coil design was efficiency in terms of power consumption. For this reason, we have designed our MPI system by keeping the number of windings [winding layers (L)] and the excitation current (I) as small as possible to obtain the required magnetic field generated by each of the coils, as shown above in Table I. The coil optimizations for each drive coil, y-axis focus coils, z-axis focus coils, and the selection field coils in terms of winding layers and excitation currents parameters are shown in Fig. 3. Electromagnetic coils inherently generate resistive heating; excessive power can reduce efficiency and compromise the long-term stability of the imaging system. To address this, the coil windings were therefore optimized to maximize magnetic field strength and field uniformity while minimizing power consumption. This balance between magnetic performance and energy efficiency ensures that the MPI system is both effective and practical for extended imaging sessions.

## III. SCANNING OF 3D SPACE

**FIG. 4.**
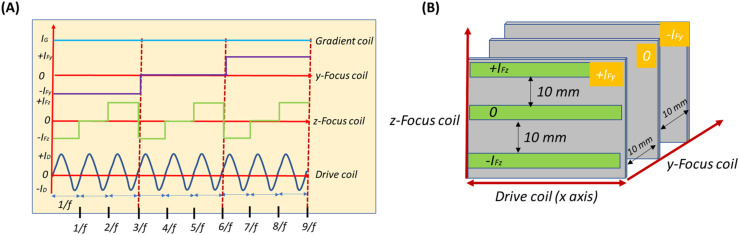
(a) Excitation currents over time for drive, y- and z-axis focus, and selection coils. (b) FFP scanning progression from 1D to 2D and finally 3D within the FOV, corresponding to the excitation currents in (a).

**FIG. 5.**
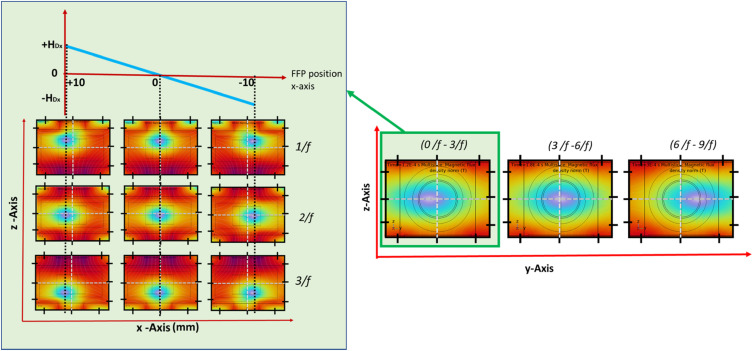
3D FFP scanning across the 20 mm^3^ FOV. Scanning begins with 1D motion along the x-axis, followed by 2D sweeps across the XZ plane. For volumetric coverage, the FFP is incrementally shifted along the y-axis, with the XZ plane scanned at each y-position. Repeating this process achieves full 3D imaging of the entire FOV.

In MPI, 3D image reconstruction relies on the controlled movement of the FFP across the imaging volume. The FFP represents the highly localized region where the nonlinear response of the MNPs is detected, and thus, accurate scanning of FFP trajectories is essential for producing high-quality images. In this work, the FFP was moved sequentially along the x-, y-, and z-axes to ensure complete spatial coverage. For our simulations, excitation signals were applied to the coils to achieve line scanning^16^ of the FFP. In order to move the FFP in the space as a function of time, the time-dependent input signals applied to the coils, specifically the AC drive coil signal and the stepwise signals of the y- and z-axis focus coils, are illustrated in Fig. 4(a), and the scanning of 2D slices to 3D FOV is shown in Fig. 4(b). For one period (1/*f*) of the drive coil signal, the FFP traced one line, and the time required to scan one line is 40 *μ*s. Then, to achieve the 2D slice scanning, the z-axis focus field coils are used with stepwise time domain current signals, where each step duration was (1/*f*) or 40 *μ*s, and the step size I_z_ in the interval of −I_z_ to I_z_. Due to computational constraints, we have only considered three steps to demonstrate the 2D x-z plane scanning. Figure 5 shows the static images of 1D line scans, then 2D x-z slice scans. The total time required to scan each 2D slice of the x-z plane is (3/*f*) or 120 *μ*s. The x-z plane’s 1D–2D slice scanning is shown in the supplementary video Fig. 6(a) (Multimedia available online) generated from the COMSOL software.

Then, from a 2D slice scanning to achieve the 3D scanning, y-axis focus field coils were used with the step duration of (3/*f*) or 120 *μ*s and the step size I_y_ in the interval of −I_y_ to +I_y_. For each y-axis position, the FFP completes the 2D slice scanning of the x-z plane. Figure 5 shows the static images of the y-z plane scanning, and the total time required to complete y-z scanning is (9/*f*) or 360 *μ*s. The y-z plane’s scanning is shown in S1 and S2 of the supplementary material generated from the COMSOL software. In the above simulations, the scanning step size is 10 mm along the z-axis (three lines) and 10 mm along the y-axis (three slices). To further improve spatial resolution by reducing the step size to 5 mm along z (five lines) and 5 mm along y (five slices), the acquisition time for a single 2D x-z plane becomes 5/*f* (200 *μ*s), and the total 3D scanning time becomes 25/*f* (1000 *μ*s, ∼1 ms). For even finer resolution, decreasing the step size to 2 mm along z (11 lines) and 2 mm along y (11 slices) increases the 2D x-z plane acquisition time to 11/*f* (440 *μ*s) and the total 3D scanning time to 121/*f* (4840 *μ*s, ∼4.84 ms).

In summary, the FFP sequentially scanned 1D lines along the x-axis, expanded into 2D x–z plane slices, and was incrementally shifted along the y-axis to achieve complete 3D volume coverage. The optimized gradient and focus fields ensured stable FFP trajectories with minimal artifacts across the 20 mm^3^ FOV. These results confirm the successful implementation of controlled 3D FFP scanning in FEM simulations using the proposed electromagnetic coil designs.

## IV. CONCLUSIONS

In this work, we have successfully demonstrated 3D FFP scanning using a MPI system prototype simulated with FEM analysis. The system was designed to operate within a 20 mm^3^ FOV, incorporating optimized coil geometries to generate the required magnetic fields: a 4 T/m selection gradient, a 20 mT drive field, and 20 mT (y-axis) and 40 mT (z-axis) focus fields. By integrating selection, drive, and focus coils into a unified FEM model and applying time-domain excitations, controlled volumetric FFP scanning was achieved. This confirmed the feasibility of a complete 3D MPI system simulation using FEM as a design and validation tool. The simulation results provide valuable insights into coil design and system-level integration, establishing a framework for advancing MPI technology. Specifically, this study highlights how FEM-based simulation can be used not only to optimize coil efficiency and field uniformity, but also to predict full 3D scanning trajectories prior to physical implementation. Future work will address practical challenges associated with clinical-scale MPI, including power efficiency, heat generation modeling, and coil cooling strategies for multiple scans. In addition, scaling the system to larger FOVs and implementing finer scanning steps will be critical for enhancing spatial resolution and translating MPI from preclinical studies to human-scale applications.

## SUPPLEMENTARY MATERIAL

A multimedia representation of the y-z plane's scanning is available in the supplementary material. See S1 for X-Z Plan FFP Scanning COMSOL Simulations and S2 for Y-Z Plan FFP Scanning COMSOL Simulations.

## Data Availability

The data that support the findings of this study are available from the corresponding authors upon reasonable request.
